# Supplementary material for: Single‐Use, Metabolite Absorbing, Resonant Transducer (SMART) Culture Vessels for Label‐Free, Continuous Cell Culture Progression Monitoring
Source: Adv Sci (Weinh). 2024 Jun 20;11(32):2401260. doi: 10.1002/advs.202401260 (PMC11348071; doi:10.1002/advs.202401260)
Supplement: Supplementary file 1 — Supporting Information [file ADVS-11-2401260-s002.pdf]

ADVANCED SCIENCE
Open Access

Supporting Information

for *Adv. Sci.*, DOI 10.1002/advs.202401260

Single-Use, Metabolite Absorbing, Resonant Transducer (SMART) Culture Vessels for Label-Free, Continuous Cell Culture Progression Monitoring

*Yee Jher Chan, Dhananjay Dileep, Samuel M. Rothstein, Eric W. Cochran and Nigel F. Reuel**

Single-use, Metabolite Absorbing, Resonant Transducer (SMART) culture vessels for label-free, continuous cell culture progression monitoring

Yee Jher Chan[1], Dhananjay Dileep[1], Samuel M. Rothstein[2], Eric W. Cochran[1], Nigel F. Reuel[1,2]*

1. Iowa State University, Chemical and Biological Engineering, Ames, IA
2. Skroot Laboratory Inc, Ames, IA

*Corresponding Author – reuel@iastate.edu

Table of Contents

Figure S1. Example of s-parameters response over frequency 3

Figure S2. Cross sectional side view of simulation of the resonant sensor system to investigate sensitivity to changing permittivity of a thin layer 4

Figure S3. Sensitivity of a wire wound resonant sensor of length 23 cm to polyethylene beads during resuspension events 5

Figure S4. Sensitivity of resonant sensor to suspending polyethylene beads 6

Figure S5. Screening of molecules containing different functional groups 7

Figure S6. SMART equilibrium response to the initial addition of culture media 8

Figure S7. SMART response to fetal bovine serum 9

Figure S8. SMART response to liposome 10

Figure S9. Differential Scanning Calorimetry and Thermogravimetric Analysis of polyacrylate after treatment with media or cells suspension 11

Figure S10. Frugal microscopic imaging system integrated with resonant sensor readout antenna 12

Figure S11. Resonant frequency profile with media exchange 13

Figure S12. Correlation between resonant frequency change and seeding concentration of HeLa cells 14

Figure S13. Resonant frequency profiles of HeLa cell culture under different concentrations of fetal bovine serum 15

Figure S14. Microscopic images of HeLa cells at different time of culture and corresponding annotated images 16

Figure S15. Correlation between resonant frequency change and seeding concentration of K562 cells 17

Figure S16. Resonant frequency profiles of Chinese hamster ovary cells in shaker flasks . 18

Table S1. Formulation of screening solutions 19

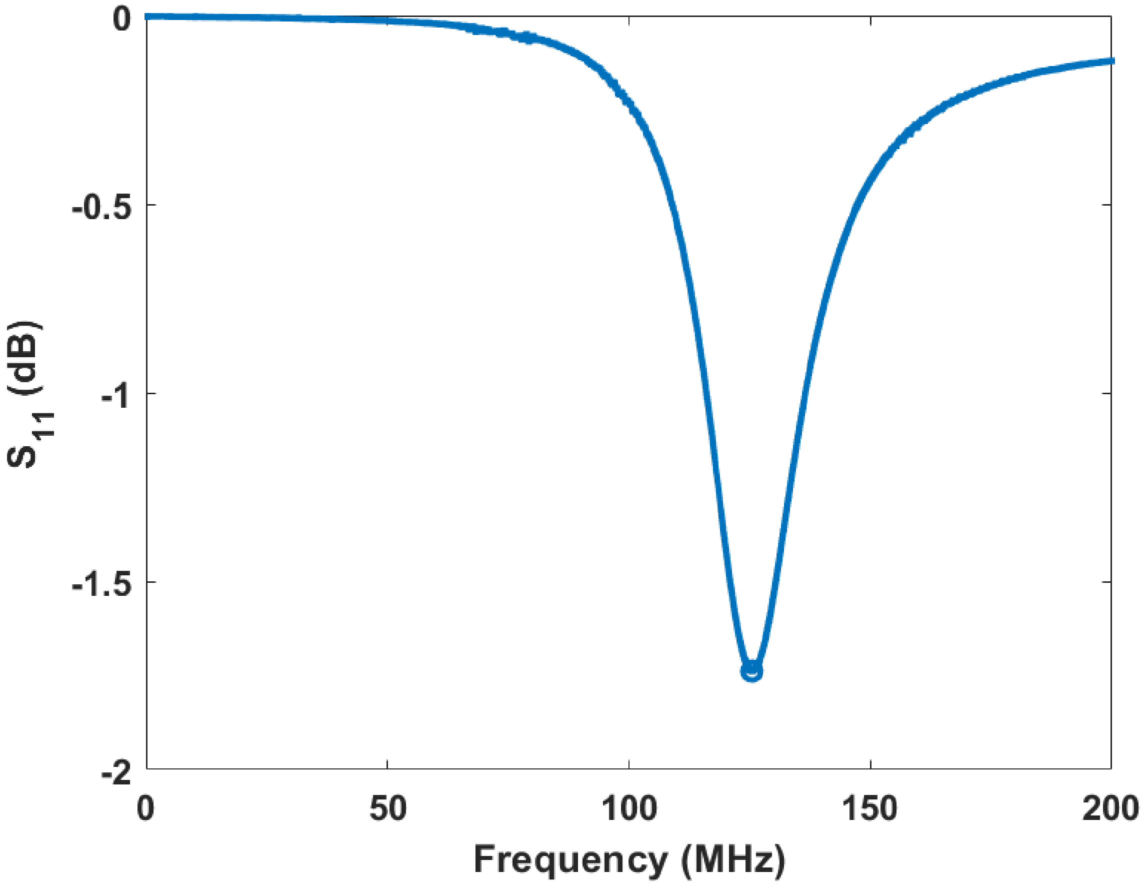

Figure S1. Example of s-parameters response over frequency. Resonant frequency is circled and determined from the spectrum minima.

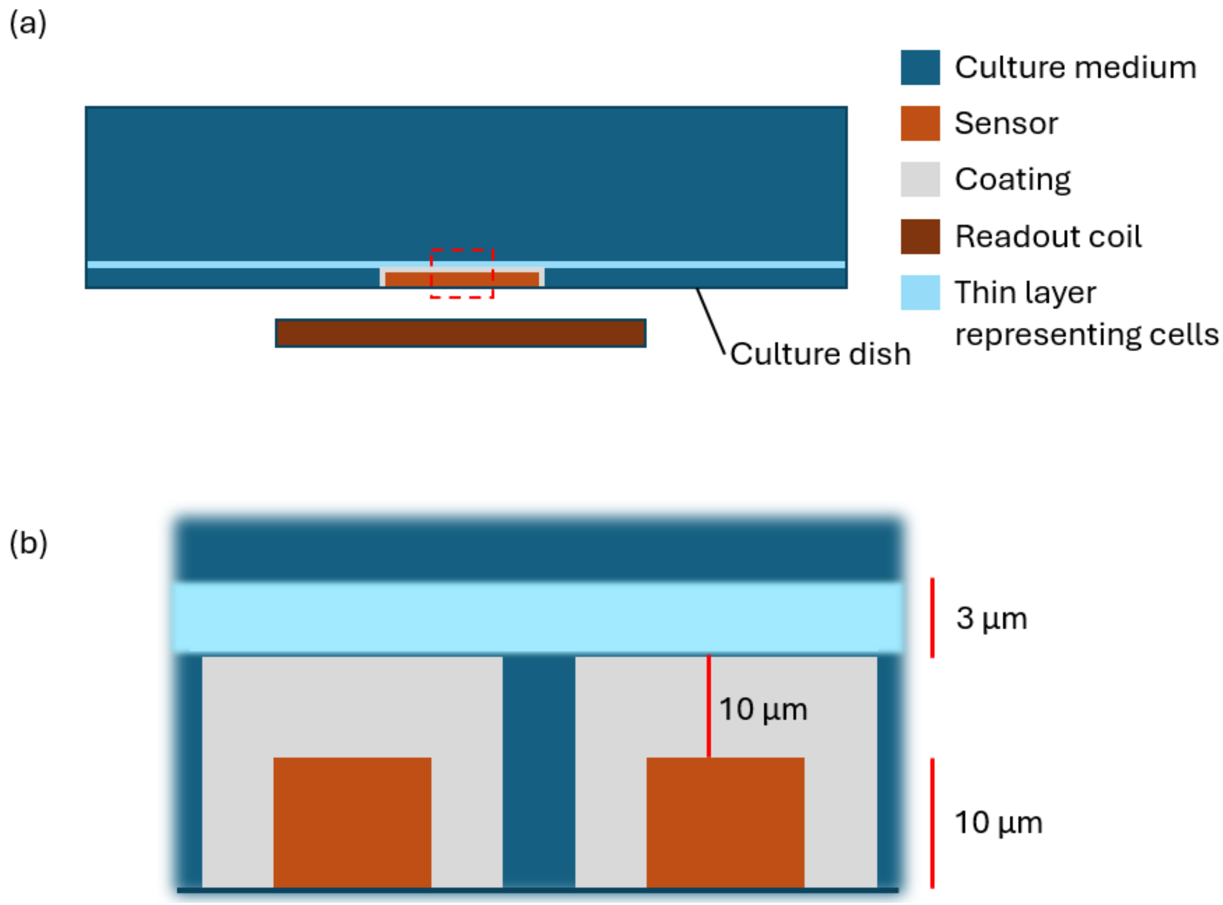

Figure S2. (a) Cross sectional side view of simulation of the resonant sensor system (see parameters below) to investigate sensitivity to changing permittivity of a thin layer (not to scale). Thin layer height: 3µm; Dish height: 10mm; Sensor height: 10µm. (b) Enlarged view of the boxed region in (a), not to scale.

Simulation parameters:

Coating permittivity: 3

Coating conductivity: 0

Thin layer permittivity: 10-90

Thin layer conductivity: 0

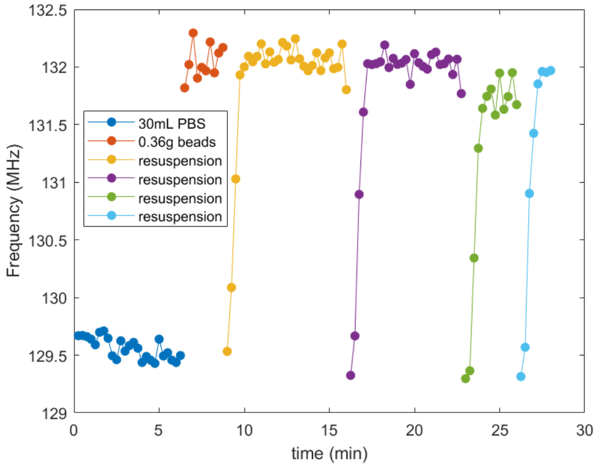

Figure S3. Sensitivity of a wire wound resonant sensor of length 23 cm to polyethylene beads during resuspension events. Resonant sensor was insulated with a 63.5µm Kapton.

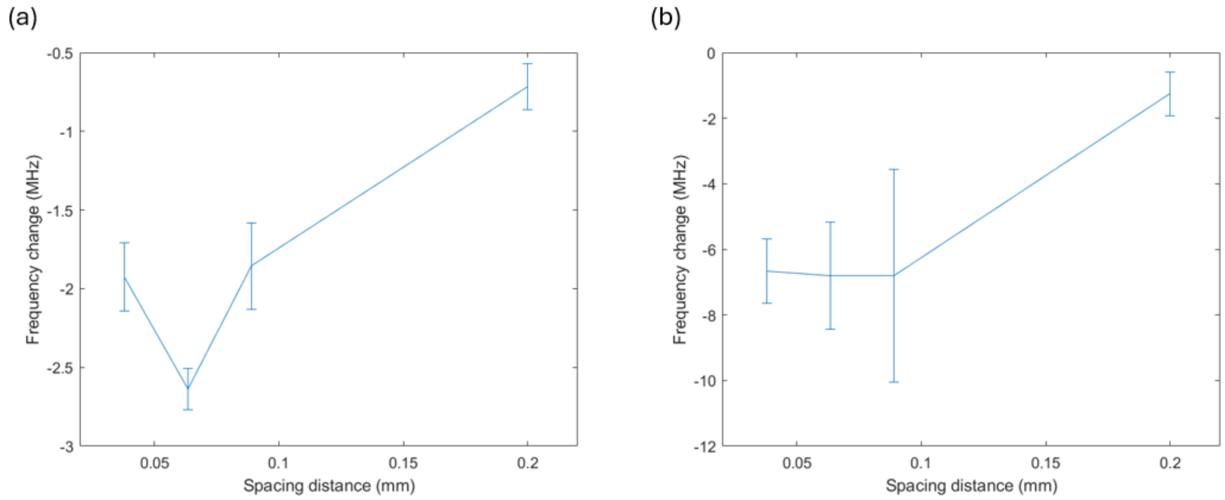

Figure S4. Sensitivity of resonant sensor to suspending polyethylene beads with (a) 23 cm wound wire and (b) 15 cm wound wire.

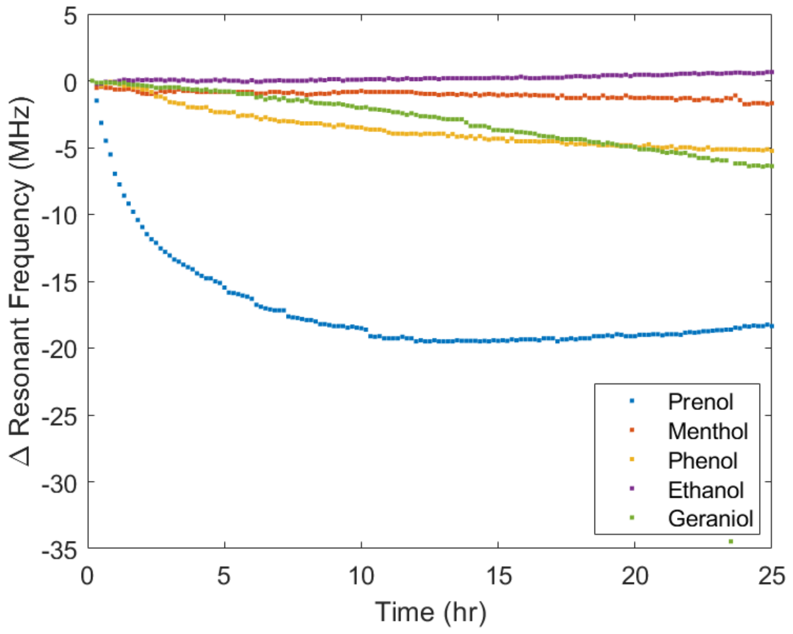

Figure S5. Screening of molecules containing different functional groups.

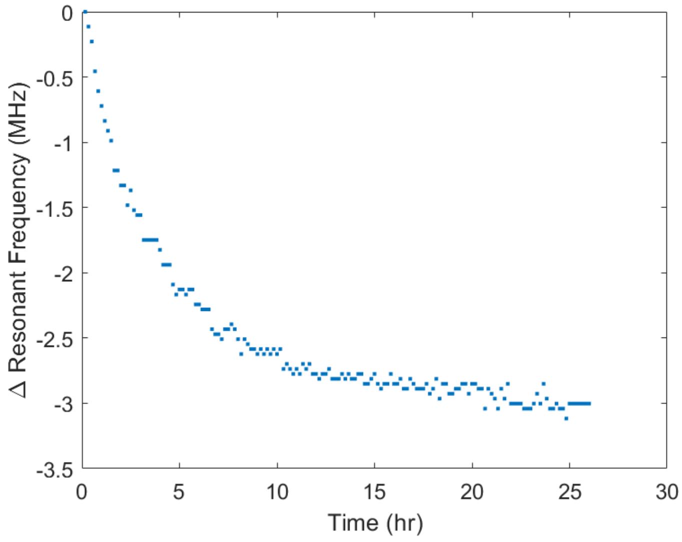

Figure S6. SMART equilibrium response to the initial addition of culture media.

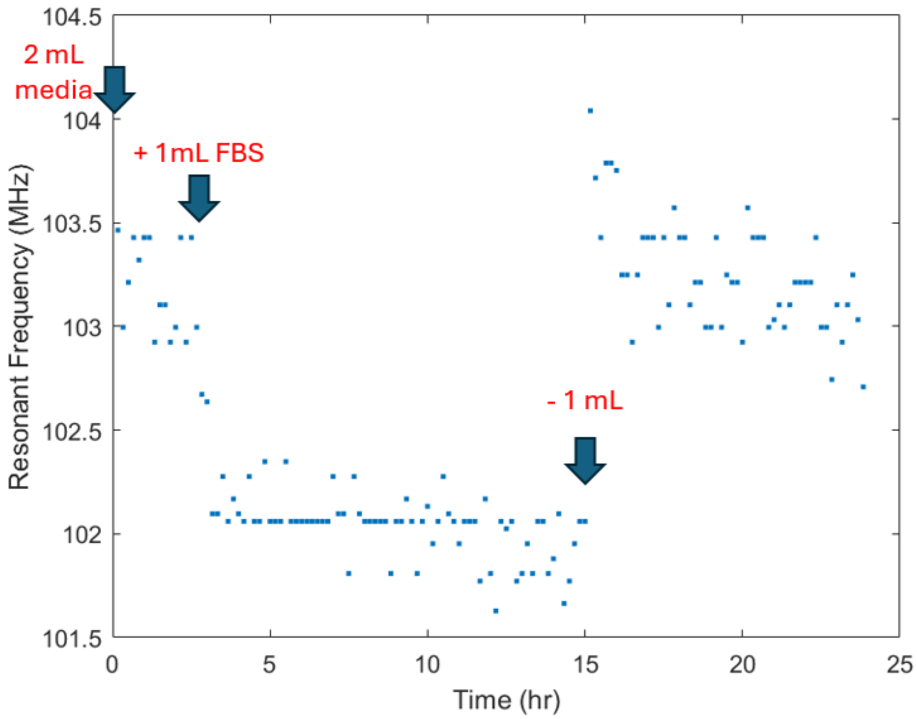

Figure S7. SMART response to fetal bovine serum (FBS). SMART was pre-equilibrated with culture media. Signal response after FBS addition is mainly due to increased media volume, as noted by return of signal upon solution removal.

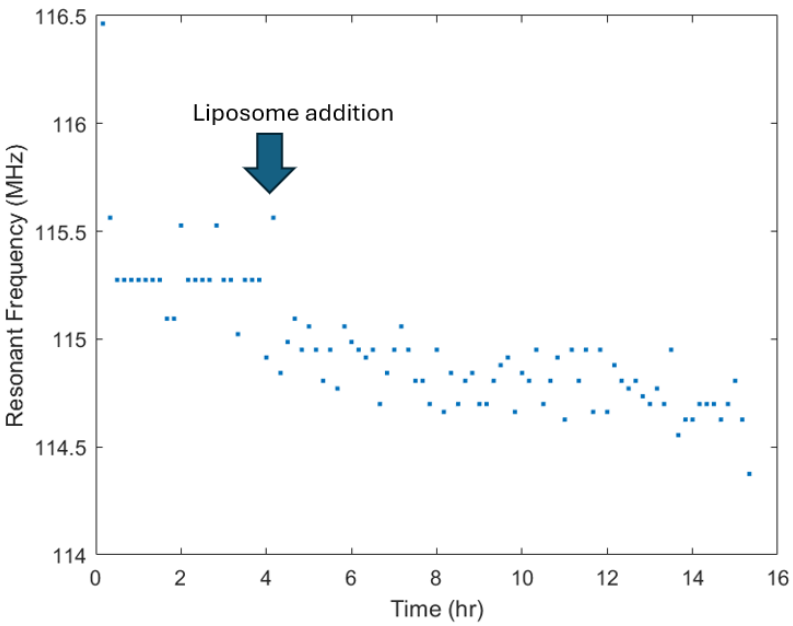

Figure S8. SMART response to liposome. SMART was pre-equilibrated with culture media.

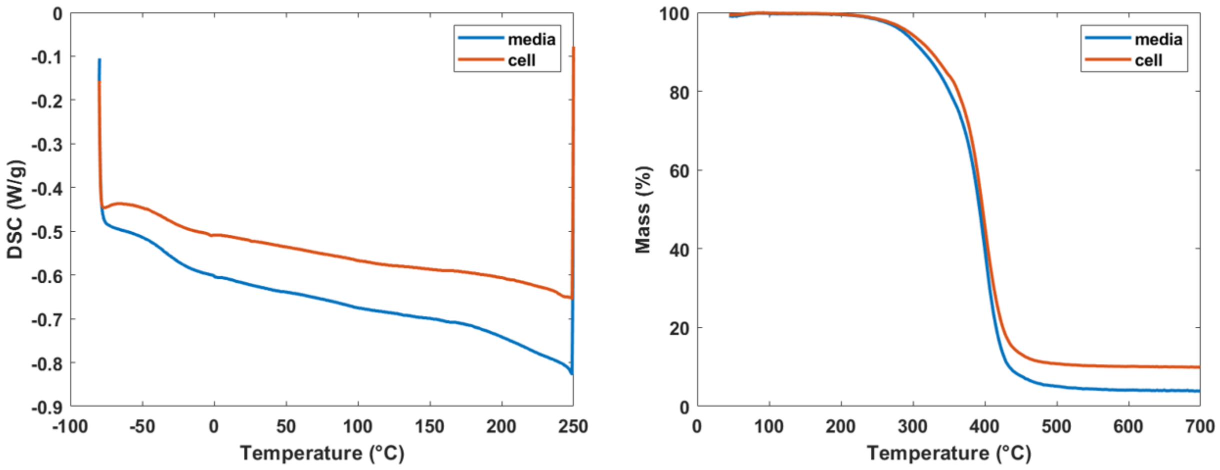

Figure S9. (Left) Differential Scanning Calorimetry and (Right) Thermogravimetric Analysis of polyacrylate after treatment with media or cells suspension.

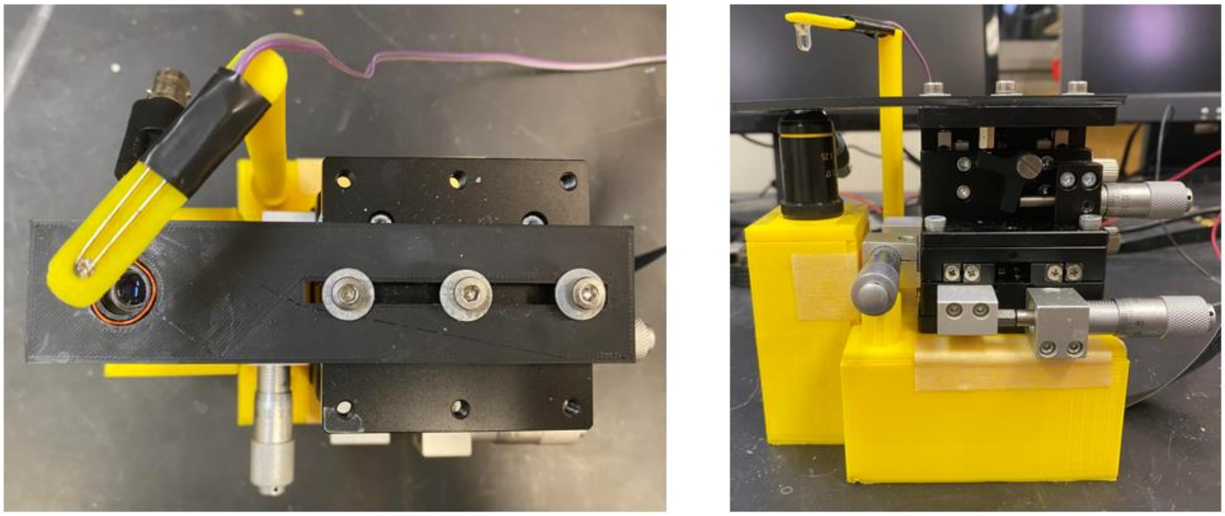

Figure S10. (Left) Top view and (Right) Side view of the frugal microscopic imaging system integrated with resonant sensor readout antenna.

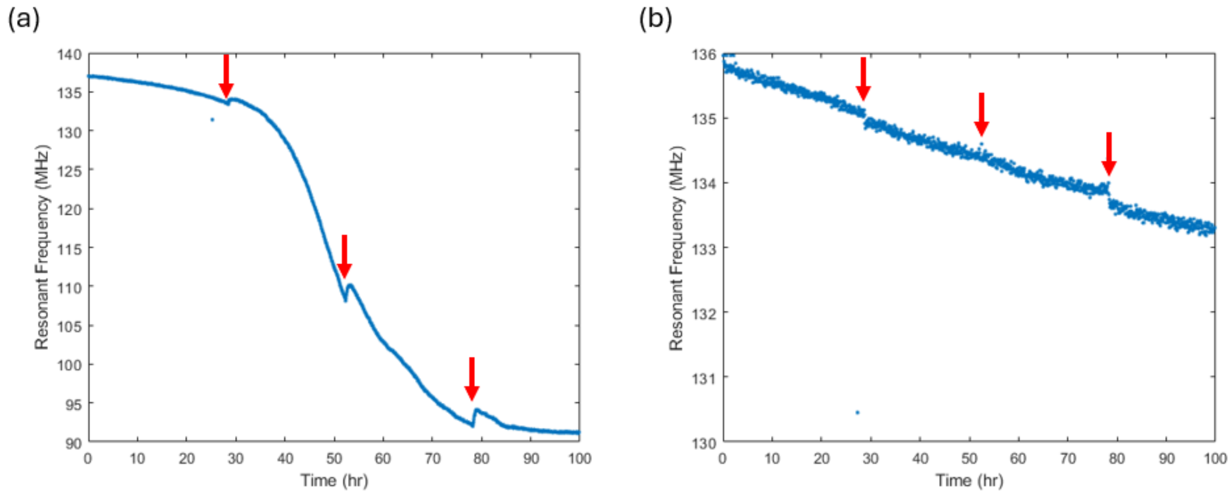

Figure S11. (a) Resonant frequency profile with media exchange at time points indicated by red arrows. (b) Resonant frequency profile of SMART dish containing only fresh media or spent media from (a) without cells.

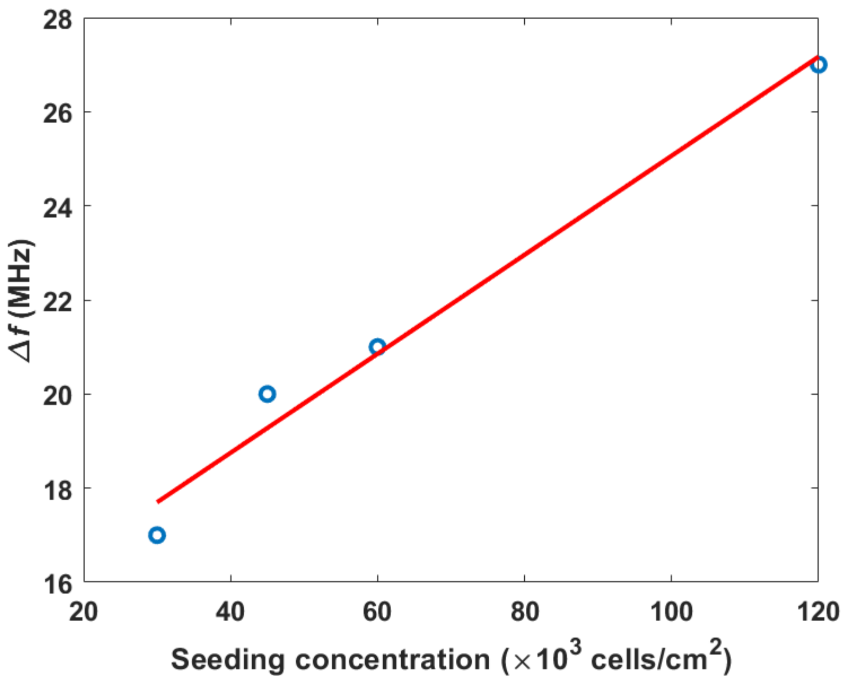

Figure S12. Correlation between resonant frequency change and seeding concentration of HeLa cells. Gain = 0.105MHz/1000cells/cm^2. R^2 = 0.98.

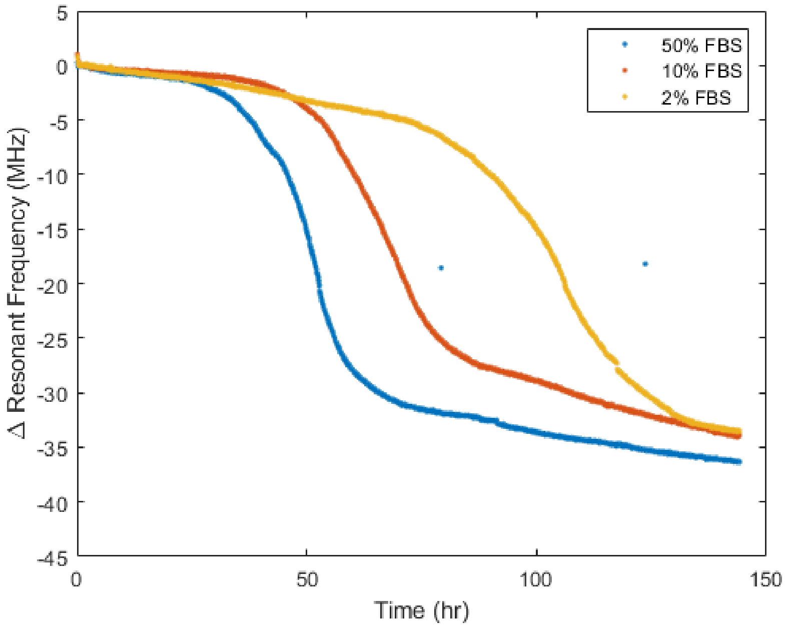

Figure S13. Resonant frequency profiles of HeLa cell culture under different concentrations of fetal bovine serum.

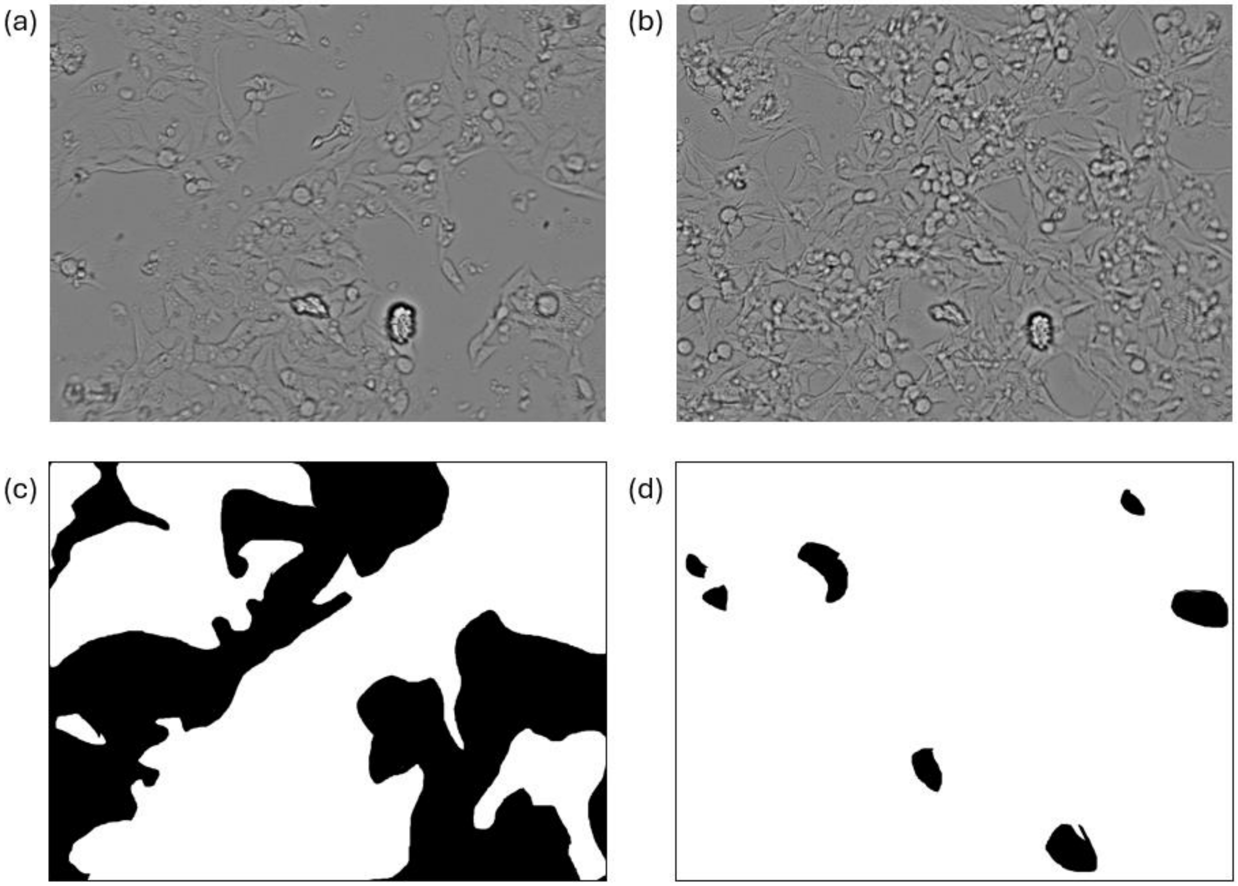

Figure S14. (a), (b) Microscopic images of HeLa cells at different time of culture and (c), (d) corresponding annotated images. White pixels represent cells whereas black pixels represent background.

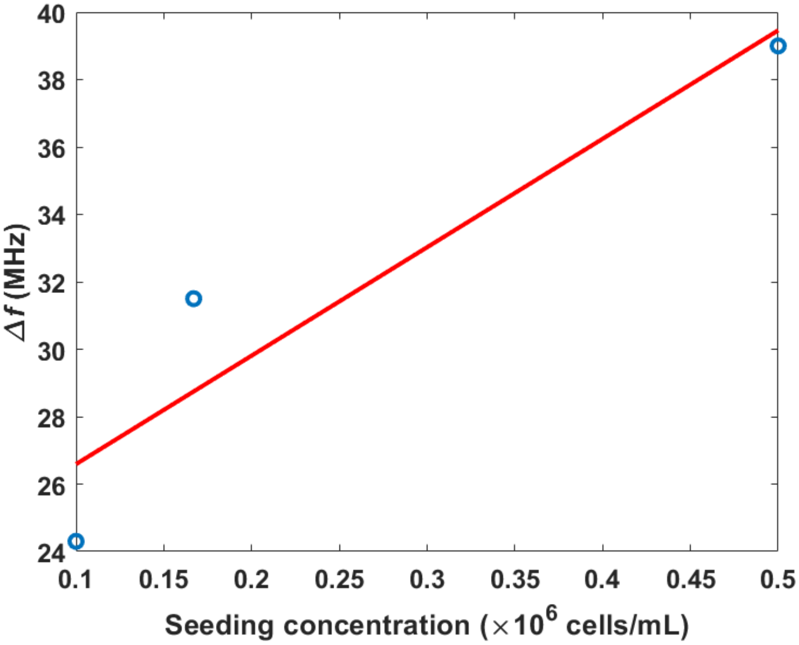

Figure S15. Correlation between resonant frequency change and seeding concentration of K562 cells. Gain = 0.032MHz/1000cells/mL. R^2 = 0.88.

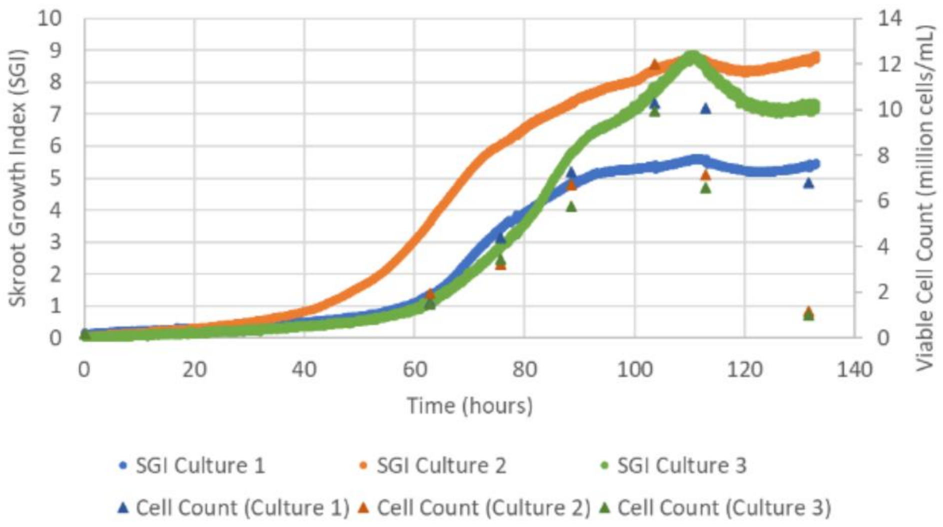

Figure S16. Resonant frequency profiles (represented in Skroot Growth Index) of Chinese hamster ovary cells in shaker flasks cultured in 80mL FreeStyle media, 37°C, 5% CO_2, and 120 RPM.

Table S1. Formulation of screening solutions

Chemical	Solvent	Concentration	Volume added (7mL total volume)
Lactic acid	-	>90 wt%	1 µL
Ethanol	-	100 %	700 µL
Phenol	Ethanol	200 mg/mL	700 µL
Quinine hemisulfate	Ethanol	8.6 mg/mL	700 µL
Pyridine hydrochloride	Ethanol	50 mg/mL	700 µL
Trigonelline hydrochloride	Water	50 mg/mL	700 µL
Hydroxyanthraquinone	Ethanol	2 mg/mL	700 µL
Erythromycin	Ethanol	50 mg/mL	700 µL
Amphotericin	Water	250 µg/mL	700 µL
Menthol	Ethanol	100 mg/mL	700 µL
Geraniol	-	98%	700 µL
Prenol	-	99%	700 µL
Spent media	-	-	Replaced with 7 mL
Fetal bovine serum	-	-	1 mL
Liposome	Phosphate-buffered saline	4:1 Dipalmitoylphosphatidylcholine/ Cholesterol	500 µL (2.5 mL total volume)
